# Supplementary figures and images for: Mucosal leishmaniasis is associated with the Leishmania RNA virus and inappropriate cutaneous leishmaniasis treatment
Source: PLoS One. 2025 Jan 24;20(1):e0317221. doi: 10.1371/journal.pone.0317221 (PMC11759362; doi:10.1371/journal.pone.0317221)

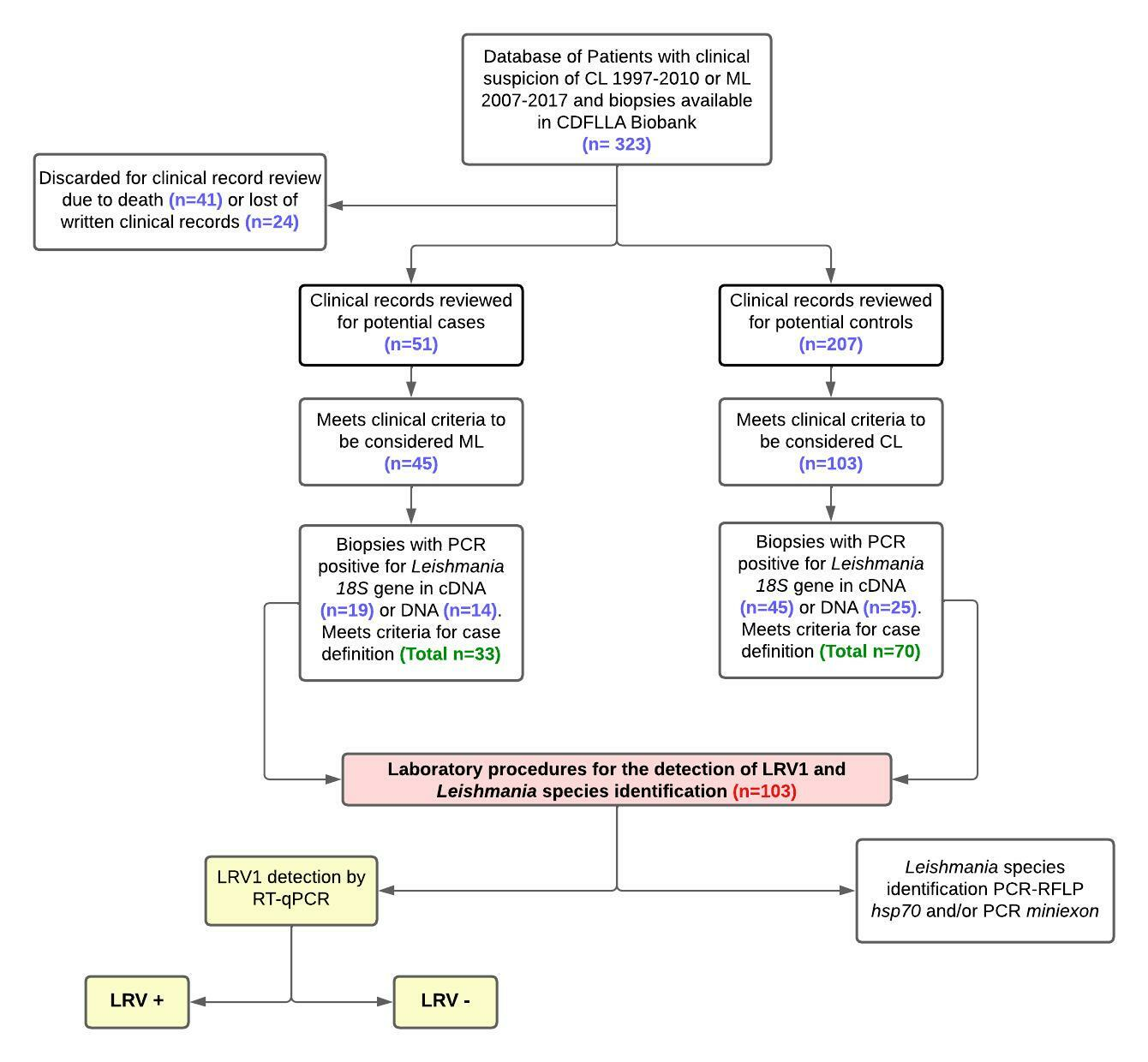

Supplement: S1 Fig — The stepwise approach describes the recruitment process and the criteria for including and classifying the patients and samples. (TIF) [file pone.0317221.s001.tif]

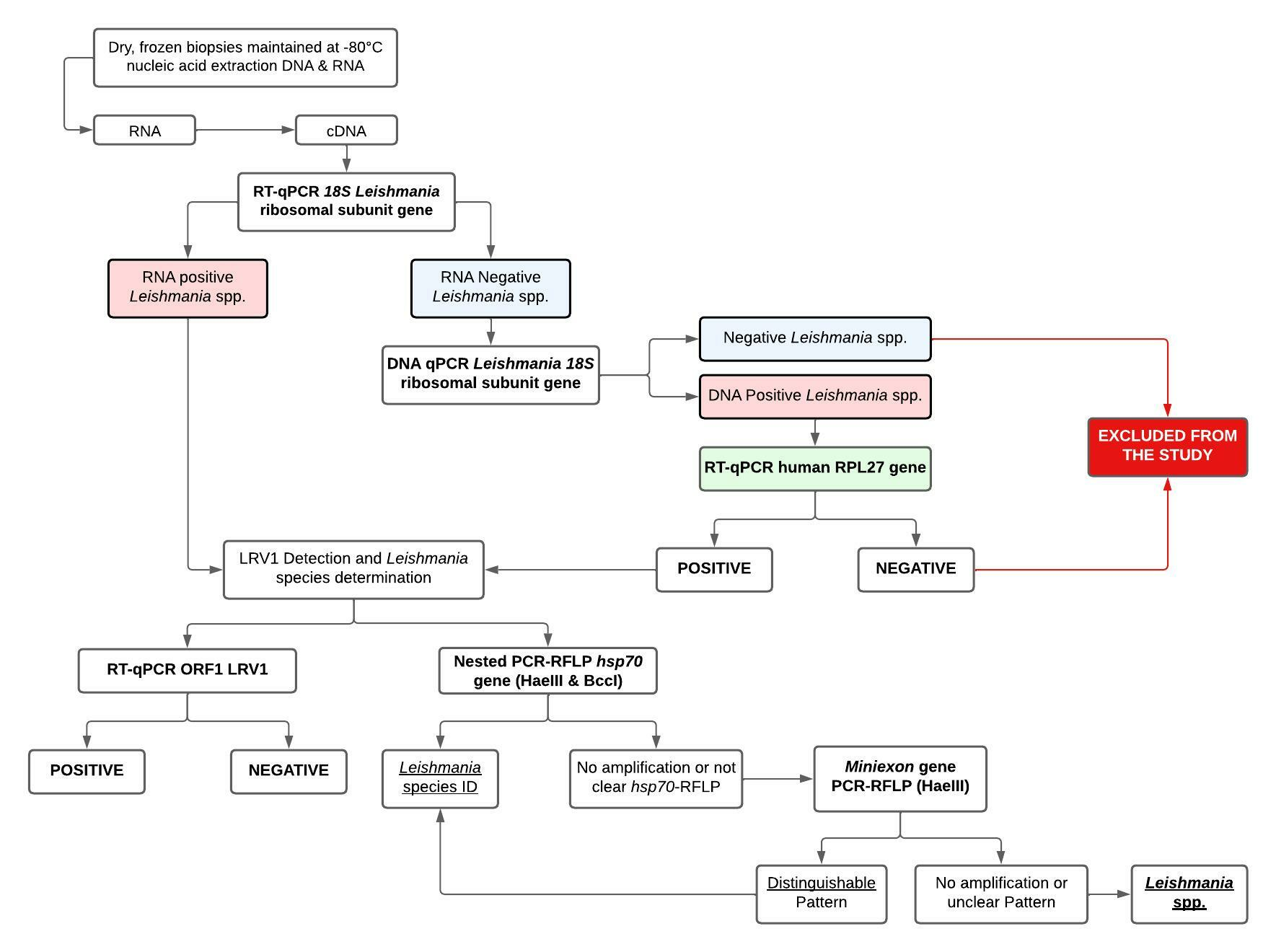

Supplement: S2 Fig — (TIF) [file pone.0317221.s002.tif]
