# Supplementary material for: Mucosal leishmaniasis is associated with the Leishmania RNA virus and inappropriate cutaneous leishmaniasis treatment
Source: PLoS One. 2025 Jan 24;20(1):e0317221. doi: 10.1371/journal.pone.0317221 (PMC11759362; doi:10.1371/journal.pone.0317221)
Supplement: S1 Table — (PDF) [file pone.0317221.s003.pdf]

Supplementary Table 1. Detailed description of selected variables per sample

Code	Clinical form	Sex	Geographic origin	Leishmania species	LRV1 status
C111	CL	F	Amazon	*L. (V.) braziliensis*	Negative
C12	CL	M	Andean	*L. (V.) panamensis*	Negative
C121	CL	M	Andean	*L. (V.) panamensis*	Negative
C125	CL	M	Amazon	*L. (V.) guyanensis*	Negative
C13	CL	M	Amazon	*L. (V.) braziliensis*	Negative
C131	CL	M	Amazon	*L. (V.) braziliensis*	Positive
C132	CL	M	Orinoco	*L. (V.) braziliensis*	Negative
C133	CL	F	Andean	*L. (V.) braziliensis*	Negative
C135	CL	M	Amazon	*L. (V.) braziliensis*	Positive
C138	CL	M	Amazon	*Leishmania spp*	Negative
C152	CL	M	Andean	*Leishmania spp*	Negative
C158	CL	M	Andean	*L. (V.) braziliensis*	Negative
C159	CL	M	NI	*L. (V.) panamensis*	Negative
C16	CL	M	Amazon	*L. (V.) braziliensis*	Negative
C168	CL	M	NI	*L. (V.) braziliensis*	Negative
C170	CL	M	Andean	*L. (V.) braziliensis*	Negative
C175	CL	F	Andean	*Leishmania spp*	Negative
C179	CL	F	Andean	*L. (V.) braziliensis*	Negative
C180	CL	M	Andean	*L. (V.) panamensis*	Negative
C185	CL	M	Amazon	*Leishmania spp*	Negative
C189	CL	M	Amazon	*Leishmania spp*	Negative
C195	CL	F	Andean	*Leishmania spp*	Positive
C197	CL	F	Andean	*L. (V.) panamensis*	Negative
C198	CL	F	Andean	*L. (V.) braziliensis*	Negative
C199	CL	M	Andean	*Leishmania spp*	Negative
C201	CL	M	Andean	*Leishmania spp*	Negative
C204	CL	M	Andean	*L. (V.) braziliensis*	Negative
C206	CL	M	Andean	*L. (V.) panamensis*	Negative
C207	CL	F	Andean	*L. (V.) panamensis*	Negative
C210	CL	M	NI	*L. (V.) braziliensis*	Negative
C218	CL	M	Pacific	*L. (V.) braziliensis*	Negative
C22	CL	F	Andean	*Leishmania spp*	Negative
C220	CL	M	Orinoco	*L. (V.) braziliensis*	Negative
C228	CL	M	NI	*L. (V.) braziliensis*	Negative
C23	CL	M	Orinoco	*L. (V.) braziliensis*	Negative
C234	CL	F	Andean	*L. (V.) braziliensis*	Negative
C236	CL	F	Andean	*L. (V.) braziliensis*	Negative
C24	CL	F	Andean	*Leishmania spp*	Negative
C241	CL	M	Andean	*L. (V.) panamensis*	Negative

Supplementary Table 1. Detailed description of selected variables per sample

Code	Clinical form	Sex	Geographic origin*	Leishmania species	LRV1 status
C245	CL	M	Amazon	*Leishmania spp*	Negative
C246	CL	M	Orinoco	*L. (V.) braziliensis*	Positive
C247	CL	M	Amazon	*L. (V.) braziliensis*	Negative
C249	CL	M	Amazon	*L. (V.) braziliensis*	Negative
C255	CL	F	Andean	*L. (V.) braziliensis*	Negative
C264	CL	M	Caribbean	*L. (V.) braziliensis*	Negative
C266	CL	F	Andean	*L. (V.) braziliensis*	Negative
C268	CL	M	Andean	*L. (V.) panamensis*	Negative
C269	CL	M	Andean	*Leishmania spp*	Negative
C278	CL	M	Orinoco	*Leishmania spp*	Negative
C28	CL	M	Orinoco	*L. (V.) braziliensis*	Negative
C288	CL	F	Andean	*Leishmania spp*	Negative
C292	CL	M	Orinoco	*L. (V.) braziliensis*	Negative
C293	CL	M	Orinoco	*Leishmania spp*	Negative
C3	CL	M	Andean	*Leishmania spp*	Negative
C302	CL	M	Orinoco	*Leishmania spp*	Negative
C316	CL	M	Amazon	*L. (V.) braziliensis*	Negative
C317	CL	M	Amazon	*L. (V.) braziliensis*	Negative
C319	CL	M	Andean	*L. (V.) braziliensis*	Negative
C32	CL	M	Amazon	*L. (V.) braziliensis*	Positive
C329	CL	M	Andean	*L. (V.) panamensis*	Negative
C331	CL	M	Pacific	*L. (V.) braziliensis*	Positive
C343	CL	F	Andean	*L. (V.) braziliensis*	Negative
C352	CL	M	Andean	*L. (V.) braziliensis*	Negative
C36	CL	M	Andean	*L. (V.) braziliensis*	Negative
C39	CL	F	Andean	*L. (V.) panamensis*	Negative
C45	CL	M	Orinoco	*L. (V.) braziliensis*	Negative
C63	CL	M	Orinoco	*L. (V.) braziliensis*	Negative
C64	CL	F	Andean	*L. (V.) braziliensis*	Negative
C77	CL	M	Andean	*Leishmania spp*	Negative
C87	CL	M	Orinoco	*Leishmania spp*	Negative
M293	ML	M	Orinoco	*Leishmania spp*	Positive
M312	ML	M	Andean	*Leishmania spp*	Positive
M313	ML	F	Andean	*L. (V.) braziliensis*	Positive
M321	ML	M	Andean	*L. (V.) braziliensis*	Positive
M322	ML	F	Andean	*L. (V.) panamensis*	Negative
M325	ML	M	Orinoco	*L. (V.) braziliensis*	Positive
M326	ML	M	Andean	*L. (V.) panamensis*	Negative
M333	ML	F	Andean	*L. (V.) braziliensis*	Positive

Supplementary Table 1. Detailed description of selected variables per sample

**Code**	**Clinical form**	**Sex**	**Geographic origin***	**Leishmania species**	**LRV1 status**
M338	ML	M	Orinoco	*L. (V.) braziliensis*	Positive
M339	ML	M	Amazon	*L. (V.) braziliensis*	Positive
M340	ML	M	Caribbean	*L. panamensis /guyanensis*	Negative
M343	ML	M	Andean	*L. (V.) panamensis*	Negative
M347	ML	M	Amazon	*L. (V.) braziliensis*	Negative
M348	ML	M	Orinoco	*L. (V.) braziliensis*	Negative
M352	ML	M	Orinoco	*L. (V.) braziliensis*	Negative
M353	ML	F	Orinoco	*L. (V.) braziliensis*	Negative
M354	ML	M	Amazon	*L. (V.) braziliensis*	Negative
M355	ML	F	Andean	*L. (V.) panamensis*	Positive
M357	ML	M	Orinoco	*L. (V.) panamensis*	Negative
M360	ML	M	Orinoco	*Leishmania spp*	Negative
M361	ML	F	Andean	*L. (V.) panamensis*	Positive
M362	ML	M	Amazon	*L. (V.) braziliensis*	Negative
M364	ML	M	Amazon	*L. (V.) guyanensis*	Negative
M365	ML	M	Andean	*L. (V.) braziliensis*	Negative
M368	ML	M	Andean	*L. (V.) braziliensis*	Negative
M372	ML	M	Andean	*L. (V.) braziliensis*	Negative
M374	ML	F	Andean	*Leishmania spp*	Negative
M375	ML	M	Amazon	*L. (V.) braziliensis*	Negative
M376	ML	M	Andean	*L. (V.) braziliensis*	Negative
M377	ML	F	Andean	*L. (V.) braziliensis*	Positive
M380	ML	F	Orinoco	*L. (V.) braziliensis*	Negative
M381	ML	M	Orinoco	*L. (V.) braziliensis*	Negative
M383	ML	M	Andean	*L. (V.) braziliensis*	Negative

NI, No information regarding the geographical origin of the infection
