# Supplementary material for: Mucosal leishmaniasis is associated with the Leishmania RNA virus and inappropriate cutaneous leishmaniasis treatment
Source: PLoS One. 2025 Jan 24;20(1):e0317221. doi: 10.1371/journal.pone.0317221 (PMC11759362; doi:10.1371/journal.pone.0317221)
Supplement: S2 Table — (PDF) [file pone.0317221.s004.pdf]

Supplementary Table 2 . qPCR, RT-qPCR and PCR data for LRV1 detection following the stepwise approach.

A	B	C	D	E	F		H	I
ID	Target 18S *Leishmania spp.* *	Target RPL27 -RT-qPCR**			Target LRV RT-qPCR			RPL27 control**
		Average Ct	SD	Tm(C°)	Average Ct	SD	Tm(C°)	
M313	RNA positive	NA	NA	NA	35,89	0,512	79,67	NA
M361	RNA positive	NA	NA	NA	35,26	0,826	79,48 - 79,67	NA
M362	RNA positive	NA	NA	NA	UND	NA	61,72 - 61,91	NA
M372	RNA positive	NA	NA	NA	UND	NA	61,53 - 62,10	NA
M375	RNA positive	NA	NA	NA	UND	NA	61,55 - 61,74	NA
M377	RNA positive	NA	NA	NA	32,65	0,386	79,67 - 79,86	NA
M380	RNA positive	NA	NA	NA	UND	NA	61,72 - 61,91	NA
M321	RNA positive	NA	NA	NA	35,77	0,204	79,59 - 79,77	NA
M325	RNA positive	NA	NA	NA	34,92	0,676	79,59 - 79,77	NA
M338	RNA positive	NA	NA	NA	35,77	0,135	79,40 - 79,77	NA
M339	DNA positive	37,2	1,033	82,27	34,12	0,377	79,77 - 79,96	NA
M347	DNA positive	33,97	0,482	77 - 77,19	UND	NA	61,55 - 62,15	C+ Exp1
M352	DNA positive	29,08	0,109	77,19 - 76,80	UND	NA	61,55	C+ Exp1
M364	DNA positive	28,35	0,083	77,38 - 77,58	UND	NA	61,55 - 61,74	C+ Exp1
M365	DNA positive	35,326	0,131	76,95 - 77,14	UND	NA	61,36 - 61,49	C+ Exp3
M374	DNA positive	33,521	0,261	77,34	UND	NA	61,55 - 61,74	C+ Exp3
M376	DNA positive	32,755	0,61	77,34	UND	NA	61,16 - 61,55	C+ Exp3
M381	DNA positive	32,85	0,236	76,95 - 77,53	UND	NA	61,36 - 61,55	C+ Exp3
M360	DNA positive	35,97	0,074	77,09	UND	NA	62,13 - 62,32	C+ Exp2
M368	DNA positive	33,89	0,146	76,90 - 77,09	UND	NA	61,94 - 62,32	C+ Exp2
M383	DNA positive	36,14	0,066	77,28 - 77,47	UND	NA	61,94 - 62,13	C+ Exp2
**M293**	RNA positive	NA	NA	NA	33,65	0,514	81,31	NA
M312	RNA positive	NA	NA	NA	35.54	0.350	80	NA
M322	DNA positive	30.44	0.046	77.19	UND	NA	61.92	C+ Exp1
M326	RNA positive	NA	NA	NA	UND	NA	NA***	NA
M333	RNA positive	NA	NA	NA	31.86	0.339	79.5	NA
M340	RNA positive	NA	NA	NA	UND	NA	NA***	NA
M343	RNA positive	NA	NA	NA	UND	NA	NA***	NA
M348	RNA positive	NA	NA	NA	UND	NA	NA***	NA
M353	DNA positive	31,14	3,705	82,27 -82,85	UND	NA	61,73 - 61,92	NA
M354	RNA positive	NA	NA	NA	UND	NA	NA	NA
M355	RNA positive	NA	NA	NA	37,4	1,238	79,78 - 79,97	NA
M357	DNA positive	28,97	0,083	77,19 - 77,38	UND	NA	61,92 - 62,11	C+ Exp1
C32	RNA positive	NA	NA	NA	36,91	0,69	79,76 - 81,89	NA
C13	RNA positive	NA	NA	NA	UND	NA	61,55	NA
C180	RNA positive	NA	NA	NA	UND	NA	61,74	NA
C199	RNA positive	NA	NA	NA	UND	NA	61,74 - 61,94	NA
C255	RNA positive	NA	NA	NA	UND	NA	61,74 - 61,94	NA
C246	RNA positive	NA	NA	NA	35,60	0,868	82,66 - 82,47	NA
C241	RNA positive	NA	NA	NA	UND	NA	61,74 - 61,94	NA
C206	RNA positive	NA	NA	NA	UND	NA	61,74	NA
C228	RNA positive	NA	NA	NA	UND	NA	61,74 - 61,94	NA
C264	RNA positive	NA	NA	NA	UND	NA	61,74 - 61,94	NA
C247	RNA positive	NA	NA	NA	UND	NA	61,74 - 61,94	NA
C269	RNA positive	NA	NA	NA	UND	NA	61,74 - 61,93	NA
C168	RNA positive	NA	NA	NA	UND	NA	61,74 - 61,93	NA
C132	RNA positive	NA	NA	NA	UND	NA	61,74 - 62,12	NA
C125	RNA positive	NA	NA	NA	UND	NA	61,74 - 61,93	NA
C268	RNA positive	NA	NA	NA	UND	NA	61,74 - 62,12	NA
C292	RNA positive	NA	NA	NA	UND	NA	61,74 - 62,54	NA
C329	RNA positive	NA	NA	NA	UND	NA	61,74 - 61,93	NA
C331	RNA positive	NA	NA	NA	35,56	0,605	82,02 - 82,21	NA
C319	RNA positive	NA	NA	NA	UND	NA	61,74 - 61,93	NA
C121	RNA positive	NA	NA	NA	UND	NA	61,74 - 61,93	NA
C288	RNA positive	NA	NA	NA	UND	NA	61,74	NA
C39	RNA positive	NA	NA	NA	UND	NA	61,74 - 61,93	NA
C133	RNA positive	NA	NA	NA	UND	NA	61,74 - 61,93	NA
C207	RNA positive	NA	NA	NA	UND	NA	61,74 - 61,93	NA
C3	RNA positive	NA	NA	NA	UND	NA	61,74 - 61,93	NA
C179	RNA positive	NA	NA	NA	UND	NA	61,93	NA
C45	RNA positive	NA	NA	NA	UND	NA	61,74 - 61,93	NA
C195	RNA positive	NA	NA	NA	36,62	0,152	79,89	NA
C16	RNA positive	NA	NA	NA	UND	NA	61,74 - 61,93	NA
C135	RNA positive	NA	NA	NA	38,93	0,552	81,63 - 82,02	NA
C249	RNA positive	NA	NA	NA	UND	NA	61,74	NA
C278	DNA positive	31,08	0,223	77,38	UND	NA	61,92	C+ Exp4
C266	DNA positive	29,77	0,202	77 - 77,19	UND	NA	61,80 - 62,13	C+ Exp4
C28	DNA positive	27,17	0,119	77 - 77,58	UND	NA	81,92 - 62,11	C+ Exp4
C218	DNA positive	29,36	0,129	77,38	UND	NA	61,72 - 61,92	C+ Exp4
C23	DNA positive	34,66	0,305	77 - 77,58	UND	NA	61,80 - 61,92	C+ Exp4
C201	DNA positive	31,05	0,122	77,58	UND	NA	61,92 - 62,11	C+ Exp4
C198	DNA positive	30,14	0,24	77,19 - 77,38	UND	NA	61,92 - 62,30	C+ Exp4
C36	DNA positive	33,564	0,305	77,58	UND	NA	61,92 - 62,11	C+ Exp4
C63	DNA positive	32,16	0,138	77 - 77,38	UND	NA	61,92 - 61,97	C+ Exp4
C24	DNA positive	31,13	0,126	77,24	UND	NA	61,72 - 61,92	C+ Exp5
C245	DNA positive	29,35	0,158	77,05 - 77,24	UND	NA	61,72 - 62,11	C+ Exp5
C317	RNA positive	NA	NA	NA	UND	NA	62,32	NA
C210	DNA positive	30,06	0,105	77,05 - 77,24	UND	NA	62,32	C+ Exp5
C220	DNA positive	27,96	0,249	77,43	UND	NA	61,93 - 62,12	C+ Exp5
C189	DNA positive	28,23	0,228	77,05 - 77,43	UND	NA	61,93 - 62,51	C+ Exp5
C343	DNA positive	25,27	0,054	77,43 -77,63	UND	NA	62,11 -62,30	C+ Exp5
C352	DNA positive	30,53	0,128	77,24 -77,43	UND	NA	61,93 - 63,28	C+ Exp5
C302	DNA positive	28,74	0,058	77,43 - 77,63	UND	NA	61,93	C+ Exp5
C12	RNA positive	NA	NA	NA	UND	NA	61,93 - 62,12	NA
C87	RNA positive	NA	NA	NA	UND	NA	61,93 - 62,12	NA
C138	RNA positive	NA	NA	NA	UND	NA	61,93 - 62,12	NA
C152	RNA positive	NA	NA	NA	UND	NA	62,12	NA
C234	RNA positive	NA	NA	NA	UND	NA	62,12	NA
C204	RNA positive	NA	NA	NA	UND	NA	61,93 - 62,12	NA
C197	RNA positive	NA	NA	NA	UND	NA	61,64 - 61,93	NA
C175	RNA positive	NA	NA	NA	UND	NA	61,97 - 67,73	NA
C158	RNA positive	NA	NA	NA	UND	NA	61,93 - 62,12	NA
C159	RNA positive	NA	NA	NA	UND	NA	61,93	NA
C170	RNA positive	NA	NA	NA	UND	NA	62,12	NA
C316	RNA positive	NA	NA	NA	UND	NA	61,93 - 62,12	NA
C22	DNA positive	32,78	0,337	77,19 - 77,38	UND	NA	61,73	C+ Exp6
C64	DNA positive	30,74	0,102	77,19 - 77,38	UND	NA	61,54 - 61,93	C+ Exp6
C77	DNA positive	34,82	0,809	77,19 - 77,38	UND	NA	61,73 - 61,93	C+ Exp6
C111	DNA positive	24,8	0,043	77,39 - 77,58	UND	NA	61,54 - 62,13	C+ Exp6
C131	DNA positive	29,2	0,247	77,19 - 77,58	36,50	0,117	79,83 - 82,72	C+ Exp6
C185	DNA positive	29,19	0,125	77,58	UND	NA	61,31 - 61,73	C+ Exp6
C236	DNA positive	29,59	0,171	77,19 - 77,38	UND	NA	61,54	C+ Exp6
C293	DNA positive	30,43	0,144	77,19 - 77,38	UND	NA	67,70 - 68,47	C+ Exp6

J	K	L	M
Controls Target LRV			
Control	Average Ct	SD	Tm°
C+	17,68	0,633	79,1
C+	15,02	0,099	79,96
C+	14,66	NA*	79,57
C+	16,64	1,015	79,76
C+	15,53	NA*	79,58
C+	16,16	0,434	79,18 - 79,37
C+	15,84	0,339	79,12 - 79,31
C+	15,78	0,719	79,12 - 79,31
C+	15,84	0,629	79,15 - 79,34
C+	14,11	0,002	79,89 - 80,09
C+	15,47	0,396	79,31
C+	14,73	0,347	79,83 - 80,03

Controls Target RPL27			
	Average Ct	SD	Tm(C°)
C+ Exp1	29.36	0.264	76.61 - 76.80
C+ Exp2	29.32	0.707	76.90 - 77.09
C+ Exp3	30.54	0.335	77.14
C+ Exp4	29.71	0.074	77
C+ Exp5	29.56	0.185	76.47
C+ Exp6	30.06	0.118	77

NA= Does not apply

UND= undetermined

* In the stepwise approach used for LRV1 detection in clinical samples, detection of parasite was performed in extracted RNA by 18S RTqPCR. When parasite was undetectable in RNA, the same target gene was used for parasite's detection in DNA.

** When the parasite was detectable in DNA but not in RNA, the RNA quality was assessed by RT-qPCR amplificaiton of the human gene ribosomal protein L27 (RPL27)

***Samples processed using Bio-Rad CFX Manager 3.1, version 3.1. The system does not report Tm under 65oC

Note: Data obtained for positive controls (LRV1 and RPL27 RT-qPCR) by each experiment are presented on columns K-M. Experiments for LRV1 detection has been grouped by color code to associated them with their respectively positive control.
Assays with samples that required ribosomal protein L27 (RPL27) confirmatory RT-qPCR are numbered on column I as C+ Exp1-6. The data obtained for positive control in each one of those experiments are presented in columns K-N, in subtable entitled:Controls Target RPL27
