# Supplementary material for: Mucosal leishmaniasis is associated with the Leishmania RNA virus and inappropriate cutaneous leishmaniasis treatment
Source: PLoS One. 2025 Jan 24;20(1):e0317221. doi: 10.1371/journal.pone.0317221 (PMC11759362; doi:10.1371/journal.pone.0317221)
Supplement: S3 Table — (PDF) [file pone.0317221.s005.pdf]

Supplementary Table 3. **A detailed description of qPCR, RT-qPCR, and PCR primary data per sample for *Leishmania* spp. detection and species classification**

Code	LRV status	Type of sample where detecting 18S	Ct 18S RNA	Ct 18S DNA	Specie by Miniexon/RFLP	Specie by hsp70/RFLP	Conclusion
M-293	Positive	RNA	28,36	NA	*No detectable*	*No detectable*	*Leishmania spp.*
M-312	Positive	RNA	33,88	NA	*No detectable*	*No detectable*	*Leishmania spp.*
M-313	Positive	RNA	43,05	NA	*N.A*	*L. (V.) braziliensis*	*L. (V.) braziliensis*
M-321	Positive	RNA	31,83	NA	*L. (V.) brazilienisis*	*No detectable*	*L. (V.) braziliensis*
M-322	Negative	DNA	Incon 41,73	33	*N.A*	*L. (V.) panamensis*	*L. (V.) panamensis*
M-325	Positive	RNA	40,12	NA	*L. (V.) brazilienisis*	*No detectable*	*L. (V.) braziliensis*
M-326	Negative	RNA	35,3	NA	*N.A*	*L. (V.) panamensis*	*L. (V.) panamensis*
M-333	Positive	RNA	36,07	NA	*N.A*	*L. (V.) braziliensis*	*L. (V.) braziliensis*
M-338	Positive	RNA	37,93	NA	*N.A*	*L. (V.) braziliensis*	*L. (V.) braziliensis*
M-339	Positive	DNA	Incon 40,27	33,2	*N.A*	*L. (V.) braziliensis*	*L. (V.) braziliensis*
M-340	Negative	RNA	30,16	NA	*L. (V.) panamensis/ L. (V.) guyanensis*	*No detectable*	*L. (V.) panamensis/ L. (V.) guyanensis complex*
M-343	Negative	RNA	25,85	NA	*N.A*	*L. (V.) panamensis*	*L. (V.) panamensis*
M-347	Negative	DNA	NA	37,43	*N.A*	*L. (V.) braziliensis*	*L. (V.) braziliensis*
M-348	Negative	RNA	33,46	NA	*N.A*	*L. (V.) braziliensis*	*L. (V.) braziliensis*
M-352	Negative	DNA	Incon 37,71	36,21	*N.A*	*L. (V.) braziliensis*	*L. (V.) braziliensis*
M-353	Negative	DNA	NA	32,22	*N.A*	*L. (V.) braziliensis*	*L. (V.) braziliensis*
M-354	Negative	RNA	32,59	NA	*N.A*	*L. (V.) braziliensis*	*L. (V.) braziliensis*
M-355	Positive	RNA	40,34	NA	*N.A*	*L. (V.) panamensis*	*L. (V.) panamensis*
M-357	Negative	DNA	Negative	38,03	*N.A*	*L. (V.) panamensis*	*L. (V.) panamensis*
M-360	Negative	DNA	Negative	37,94	*No detectable*	*No detectable*	*Leishmania spp.*
M-361	Positive	RNA	39,38	NA	*N.A*	*L. (V.) panamensis*	*L. (V.) panamensis*
M-362	Negative	RNA	41,48	NA	*N.A*	*L. (V.) braziliensis*	*L. (V.) braziliensis*
M-364	Negative	DNA	Negative	37,63	*N.A*	*L. (V.) guyanensis*	*L. (V.) guyanensis*
M-365	Negative	DNA	Negative	34,57	*N.A*	*L. (V.) braziliensis*	*L. (V.) braziliensis*
M-368	Negative	DNA	Negative	34,76	*N.A*	*L. (V.) braziliensis*	*L. (V.) braziliensis*
M-372	Negative	RNA	38,35	NA	*N.A*	*L. (V.) braziliensis*	*L. (V.) braziliensis*
M-374	Negative	DNA	Negative	37,68	*No detectable*	*No detectable*	*Leishmania spp.*
M-375	Negative	RNA/DNA	40,47	32,46	*L. (V.) brazilienisis*	*No detectable*	*L. (V.) braziliensis*
M-376	Negative	DNA	Negative	37,96	*N.A*	*L. (V.) braziliensis*	*L. (V.) braziliensis*
M-377	Positive	RNA	32,56	NA	*N.A*	*L. (V.) braziliensis*	*L. (V.) braziliensis*
M-380	Negative	RNA	40,05	NA	*N.A*	*L. (V.) braziliensis*	*L. (V.) braziliensis*
M-381	Negative	DNA	Negative	35,54	*N.A*	*L. (V.) braziliensis*	*L. (V.) braziliensis*
M-383	Negative	DNA	Negative	34,4	*N.A*	*L. (V.) braziliensis*	*L. (V.) braziliensis*
C39	Negative	RNA	30,92	18,09	*N.A*	*L. (V.) panamensis*	*L. (V.) panamensis*
C133	Negative	RNA/DNA	39,82	33,68	*N.A*	*L. (V.) braziliensis*	*L. (V.) braziliensis*
C207	Negative	RNA	32,78	NA	*N.A*	*L. (V.) panamensis*	*L. (V.) panamensis*
C278	Negative	DNA	Negative	37,97	*No detectable*	*No detectable*	*Leishmania spp.*
C3	Negative	RNA	41,42	NA	*No detectable*	*No detectable*	*Leishmania spp.*
C266	Negative	DNA	Negative	30,18	*N.A*	*L. (V.) braziliensis*	*L. (V.) braziliensis*
C179	Negative	RNA	27,95	NA	*L. (V.) brazilienisis*	*No detectable*	*L. (V.) braziliensis*
C45	Negative	RNA	43,10	NA	*L. (V.) brazilienisis*	*No detectable*	*L. (V.) braziliensis*
C28	Negative	DNA	Negative	35,80	*N.A*	*L. (V.) braziliensis*	*L. (V.) braziliensis*
C218	Negative	DNA	Negative	35,11	*N.A*	*L. (V.) braziliensis*	*L. (V.) braziliensis*

Supplementary Table 3. **A detailed description of qPCR, RT-qPCR, and PCR primary data per sample for *Leishmania* spp. detection and species classification**

C195	Positive	RNA	41,72	NA	*No detectable*	*hsp70 Positive but not distinguishing RFLP*	*Leishmania spp.*
C16	Negative	RNA	42,88	NA	*N.A*	*L. (V.) braziliensis*	*L. (V.) braziliensis*
C135	Positive	RNA	37,73	NA	*L. (V.) brazilienisis*	*No detectable*	*L. (V.) braziliensis*
C249	Negative	RNA/DNA	42,21	35,23	*N.A*	*L. (V.) braziliensis*	*L. (V.) braziliensis*
C23	Negative	DNA	Negative	32,70	*L. (V.) brazilienisis*	*No detectable*	*L. (V.) braziliensis*
C63	Negative	RNA/DNA	Positive	34,03	*L. (V.) brazilienisis*	*No detectable*	*L. (V.) braziliensis*
C32	Positive	RNA	39,09	NA	*L. (V.) brazilienisis*	*No detectable*	*L. (V.) braziliensis*
C201	Negative	DNA	Negative	35,79	*No detectable*	*hsp70 Positive but not distinguishing RFLP*	*Leishmania spp.*
C198	Negative	DNA	Negative	36,68	*No detectable*	*L. (V.) braziliensis*	*L. (V.) braziliensis*
C36	Negative	DNA	Negative	37,96	*No detectable*	*L. (V.) braziliensis*	*L. (V.) braziliensis*
C24	Negative	DNA	Negative	36,41	*No detectable*	*No detectable*	*Leishmania spp.*
C13	Negative	RNA	29,77	NA	*L. (V.) brazilienisis*	*No detectable*	*L. (V.) braziliensis*
C180	Negative	RNA	29,09	NA	*L. (V.) panamensis/ L. (V.) guyanensis*	*L. (V.) panamensis*	*L. (V.) panamensis*
C199	Negative	RNA	37,75	NA	*No detectable*	*hsp70 Positive but not distinguishing RFLP*	*Leishmania spp.*
C255	Negative	RNA	37,07	NA	*N.A*	*L. (V.) braziliensis*	*L. (V.) braziliensis*
C246	Positive	RNA	31,68	NA	*L. (V.) brazilienisis*	*No detectable*	*L. (V.) braziliensis*
C245	Negative	DNA	Negative	32,44	*Leishmania Viannia*	*No detectable*	*Leishmania spp.*
C241	Negative	RNA	43,52	NA	*L. (V.) panamensis/ L. (V.) guyanensis*	*L. (V.) panamensis*	*L. (V.) panamensis*
C206	Negative	RNA	28,67	NA	*L. (V.) panamensis/ L. (V.) guyanensis*	*L. (V.) panamensis*	*L. (V.) panamensis*
C210	Negative	DNA	Negative	31,99	*N.A*	*L. (V.) braziliensis*	*L. (V.) braziliensis*
C220	Negative	DNA	Negative	33,15	*N.A*	*L. (V.) braziliensis*	*L. (V.) braziliensis*
C228	Negative	RNA	28,53	NA	*L. (V.) brazilienisis*	*No detectable*	*L. (V.) braziliensis*
C264	Negative	RNA	38,68	NA	*N.A*	*L. (V.) braziliensis*	*L. (V.) braziliensis*
C269	Negative	RNA	41,67	NA	*No detectable*	*No detectable*	*Leishmania spp.*
C189	Negative	DNA	Negative	37,01	*No detectable*	*No detectable*	*Leishmania spp.*
C168	Negative	RNA	36,62	NA	*N.A*	*L. (V.) braziliensis*	*L. (V.) braziliensis*
C132	Negative	RNA	33,38	NA	*N.A*	*L. (V.) braziliensis*	*L. (V.) braziliensis*
C125	Negative	RNA	29,64	NA	*N.A*	*L. (V.) guyanensis*	*L. (V.) guyanensis*
C268	Negative	RNA	40,03	NA	*N.A*	*L. (V.) panamensis*	*L. (V.) panamensis*
C292	Negative	RNA	34,77	NA	*N.A*	*L. (V.) braziliensis*	*L. (V.) braziliensis*
C329	Negative	RNA	35,10	NA	*L. (V.) panamensis/ L. (V.) guyanensis*	*L. (V.) panamensis*	*L. (V.) panamensis*
C331	Positive	RNA	35,41	NA	*L. (V.) brazilienisis*	*No detectable*	*L. (V.) braziliensis*
C343	Negative	RNA/DNA	40,72	35,13	*N.A*	*L. (V.) braziliensis*	*L. (V.) braziliensis*
C352	Negative	DNA	Negative	25,93	*L. (V.) brazilienisis*	*No detectable*	*L. (V.) braziliensis*
C121	Negative	RNA	35,88	NA	*N.A*	*L. (V.) panamensis*	*L. (V.) panamensis*
C247	Negative	RNA	37,83	Indet	*N.A*	*L. (V.) braziliensis*	*L. (V.) braziliensis*
C288	Negative	RNA	40,19	NA	*No detectable*	*No detectable*	*Leishmania spp.*
C302	Negative	DNA	Negative	34,22	*No detectable*	*hsp70 Positive but not distinguishing RFLP*	*Leishmania spp.*
C319	Negative	RNA	36,88	NA	*Leishmania Viannia*	*L. (V.) braziliensis*	*L. (V.) braziliensis*
C317	Negative	RNA/DNA	36,13	36,64	*N.A*	*L. (V.) braziliensis*	*L. (V.) braziliensis*
C12	Negative	RNA	27,15	NA	*L. (V.) panamensis/ L. (V.) guyanensis*	*L. (V.) panamensis*	*L. (V.) panamensis*
C22	Negative	DNA	Negative	39,12	*No detectable*	*No detectable*	*Leishmania spp.*
C64	Negative	DNA	Negative	30,98	*N.A*	*L. (V.) braziliensis*	*L. (V.) braziliensis*
C77	Negative	DNA	Negative	38,76	*No detectable*	*No detectable*	*Leishmania spp.*
C87	Negative	RNA/DNA	36,75	35,21	*No detectable*	*No detectable*	*Leishmania spp.*
C111	Negative	DNA	Negative	31,26	*L. (V.) brazilienisis*	*No detectable*	*L. (V.) braziliensis*
C131	Positive	DNA	Negative	35,67	*N.A*	*L. (V.) braziliensis*	*L. (V.) braziliensis*

Supplementary Table 3. **A detailed description of qPCR, RT-qPCR, and PCR primary data per sample for *Leishmania* spp. detection and species classification**

C138	Negative	RNA	37.84	NA	*No detectable*	*No detectable*	*Leishmania spp.*
C152	Negative	RNA	35,94	NA	*No detectable*	*hsp70 Positive but not distinguishing RFLP*	*Leishmania spp.*
C185	Negative	DNA	Negative	34,59	*No detectable*	*No detectable*	*Leishmania spp.*
C234	Negative	RNA	37,51	NA	*N.A*	*L. (V.) braziliensis*	*L. (V.) braziliensis*
C236	Negative	DNA	Negative	29,00	*N.A*	*L. (V.) braziliensis*	*L. (V.) braziliensis*
C204	Negative	RNA	37,66	NA	*N.A*	*L. (V.) braziliensis*	*L. (V.) braziliensis*
C197	Negative	RNA	33,40	NA	*N.A*	*L. (V.) panamensis*	*L. (V.) panamensis*
C175	Negative	RNA	35,68	NA	*No detectable*	*hsp70 Positive but not distinguishing RFLP*	*Leishmania spp.*
C158	Negative	RNA	35,63	NA	*N.A*	*L. (V.) braziliensis*	*L. (V.) braziliensis*
C159	Negative	RNA	36,30	NA	*N.A*	*L. (V.) panamensis*	*L. (V.) panamensis*
C170	Negative	RNA	34,06	NA	*N.A*	*L. (V.) braziliensis*	*L. (V.) braziliensis*
C293	Negative	DNA	Negative	33,67	*No detectable*	*hsp70 Positive but not distinguishing RFLP*	*Leishmania spp.*
C316	Negative	RNA	34,30	NA	*N.A*	*L. (V.) braziliensis*	*L. (V.) braziliensis*

NA or N.A., the test was not applied to this sample.
