# Supplementary material for: Mucosal leishmaniasis is associated with the Leishmania RNA virus and inappropriate cutaneous leishmaniasis treatment
Source: PLoS One. 2025 Jan 24;20(1):e0317221. doi: 10.1371/journal.pone.0317221 (PMC11759362; doi:10.1371/journal.pone.0317221)
Supplement: Attachment — Submitted filename: Responses to the previous Editor and.pdf [file pone.0317221.s006.pdf]

Responses to the previous Editor and/or reviewer

Please find the required amendments attached. The lines referred to in the present document correspond with the numbering in the file “Revised manuscript with track changes”.

Reviewer #1: In line 74, correct the words "Treatment Failure" to lowercase.

⇒ Correction in line 76

In line 97, correct LRV to LRV1.

⇒ Correction in line 99

In line 164, describe the quantities of total RNA used in cDNA synthesis. Were they quantified? How?

⇒ Correction in line 168

In line 172, correct from Bio-Rad to (Bio-Rad).

⇒ Correction in line 177

In line 207, remove a comma after cutaneous lesion.

⇒ Correction in line 212

In Figure 1 and its caption, correct LRV to LRV1.

⇒ Correction in line 250

Reviewer #1: The authors presented conclusions along with a brief discussion of the main findings.
In line 276, it's important to note that the lower frequency of LRV1 found compared to other Latin American countries is due to the fact that few cases of L. guyanensis are reported in the study area, and it has already been demonstrated that L. panamensis harbors LRV1 less frequently (ref 38).

⇒ Response:

The referee is right about the apparent low frequency of LRV in *L.(V) panamensis*. However, this is not why we report lower frequencies of LRV1 infection in our samples, given that almost 60% of our samples correspond with *L.(V) braziliensis*. The studies cited by us in the mentioned paragraph have similar *L.(V) braziliensis* frequencies to our

work or even higher proportions of samples infected with this species, namely, Cantanhêde LM,2015 55%; Ito MM, 2015, 81% and in Kariyawasam R,2020 100%.

Therefore, there is no reason to mention parasite species that are not well represented in our study or the studies we are comparing.

In line 297, explore more references or rewrite the phrase "Many experimental studies...".

⇒ Correction in line 305

In line 302, correct LRV-1 to LRV1.

⇒ Correction in line 310

In line 303, correct Ito MM to Ito.

⇒ Correction in line 312

Lines 315 to 317, the discussion needs better substantiation, including references.

⇒ The discussion was amplified in the paragraph between lines 325-3301 with the inclusion of new references (39-41)

Reviewer #1: I suggest that Figure 1 be placed as the main figure of the article. It would be interesting to include in Fig S1 a map highlighting the origin locations of the patients such as Orinoco, Amazon.

⇒ The figure was included as Fig. S2 and referred to in the main text on lines 247-48

Abstract: In the methods and results sections, replace LRV with LRV1, as the research was conducted to search for LRV1.

⇒ Corrections in lines: 32,36,38 and 41

Rewrite the sentence in line 49: "For a long time, it has been known that some parasite strains can be infected with a virus identified as Leishmaniavirus."

⇒ All the author Summary was removed.

When citing subgenres, correct all italicized parentheses.

⇒ Corrections were made all along the manuscript.

Reviewer #1: The article overall explores the relationship between the presence of LRV1 and ML cases. The data are relevant and consistent with the literature. However, a significant finding

regarding complete treatment as a protective factor against ML occurrence should be further explored. This finding could be emphasized in the article's title, but it should contain more discussion and references on this point, which, while not novel, is important for public policy measures.

⇒ Response: The title of the manuscript was modified, and the discussion was amplified in the paragraph between lines 325-331 with the inclusion of new references (39-41).

In the discussion/Conclusion, it is important to emphasize that, due to the nature of data obtained from cryopreserved samples over many years, proper precautions were taken to avoid false-negative results, such as the amplification of Leishmania genes in the cDNA samples. This aspect was very vague in the methodology, and the attached spreadsheet is confusing.

⇒ A sentence in lines 172-173 was introduced to emphasize this topic.

Reviewer #2: The manuscript by Pazmino et al evaluate the association LVR1 leishmania virus with the mucosal lesions caused by the parasite infection. The thematic is of importance for the specific field and for the cutaneous leishmaniasis area. The manuscript is well written and conducted with scientific standards. The manuscript reinforces the association between LRV1 and ML, highlighting the potential role of LRV as a risk factor for ML.
Points for consideration:

1) The dependance on retrospective data from clinical records may introduce potential biases and limitations, such as incomplete or inaccurate data collection and recall bias.

⇒ The referee is right. This aspect of the study design was addressed in the methodology section on line 162 about data loss and in the discussion section on lines 327-28 and 332-33.

2) The manuscript mentions that informed consent was obtained, but it does not specify how consent was managed for patients with incomplete follow-up data.

⇒ This information is in the methods section on lines 108-112, stating that informed consent was given when the clinical biopsy was taken from the patient, authorizing “the use of the stored sample, analyzing clinical records, and updating the contact details.”

3) What is the the rationale for selecting controls from a specific timeframe only?

⇒ To follow up on the onset of mucosal manifestations, a time window of at least 8 years after the CL event was given. That consideration was probably not clear in

the original manuscript; therefore, a sentence on lines 333-35 was introduced in the discussion section.

Maria Clara Echeverry.
