# Supplementary material for: Mucosal leishmaniasis is associated with the Leishmania RNA virus and inappropriate cutaneous leishmaniasis treatment
Source: PLoS One. 2025 Jan 24;20(1):e0317221. doi: 10.1371/journal.pone.0317221 (PMC11759362; doi:10.1371/journal.pone.0317221)
Supplement: Attachment — Submitted filename: Response to Reviewers.pdf [file pone.0317221.s007.pdf]

Dear reviewers, thanks for evaluating this work. Please find below the detailed response to your observations and note that the lines referred to in this response correspond with the line numbers in the revised manuscript with Track Changes.

Reviewer #1: This is an interesting manuscript evaluating whether the presence of LRV1 is associated with mucosal manifestations (ML) of American tegumentary leishmaniasis in Colombia.

Specific points:

Abstract - lines 37-38: Please revise this sentence for clarity as follows: The overall frequency of LRV1 was 16.5% (95% CI, 10.4 – 25.12), being higher in samples from cases than controls [33.33% (95% CI, 18.89 – 51.76) vs. 8.57% (95% CI, 3.82 – 18.10)].”

⇒ The abstract was corrected in lines 37-39, affirming that:
“The frequency of LRV1 in the 103 patients was 16.5% (95% CI,10.4 – 25.12) being higher in samples from cases [33.33 % (95% CI,18.89 – 51.76) than from controls [8.57% (95% CI, 3.82 – 18.10)].”

Methods

Line 113: “therapeutic test” – which one? Please specify it.

⇒ In lines 115-116, it was clarified: “therapeutic test (Defined as the improvement of signs and symptoms one month after starting treatment with pentavalent antimonials),”

Line 117: please replace “18S ribosomal gene” with “18S ribosomal RNA gene”

⇒ Replacement is done in line 119.

Line 125: where the “parasite” > where the “Leishmania parasite”

⇒ Replacement is done in line 128.

Lines 142-144: Does the definition of complete treatment for the CL episode(s) mean that the patient completed the round(s) of treatment with a successful clinical treatment outcome (i.e. cure)? Please clarify this in the text.

⇒ Clarification was done by modifying the sentence in lines 145-148 as follows:
“Complete treatment for CL was considered when patients received a least one complete round of any of the following regimes independently from the therapeutic outcome: antimony salts (20 mg/kg/ day) for 20 days, or miltefosine (1.5 to 2.5 mg/kg/ day) for 28 days, or ketoconazole (600 mg/day) for 28 days”

Line 143: the duration of the miltefosine treatment needs to be indicated

⇒ Modification is done in line 147.

Line 174: for the multivariate model, please specify that a multiple logistic regression analysis was performed

- ⇒ Modification is done in line 183.

Results

Regarding the therapeutic schedule for the patients, did the cure rate for the CL episode(s) vary among subjects treated with meglumine antimoniate or the other drugs?

- ⇒ Unfortunately, this retrospective design with a reduced "n" for treatment regimens different from antimonials is not appropriate to describe any variation in the therapeutic outcome, and it is not the focus of the study. However, to describe the information recorded about the therapeutic response, a paragraph was inserted between lines 197-209 as follows:

"In the case group, the received treatment for the CL event was: 12 patients received meglumine antimoniate, one received miltefosine, one received ketoconazole, seven did not receive treatment because they did not have cutaneous leishmaniasis, and 12 patients were not treated with any medication. Of the treated patients in the cases group, 12 showed skin lesion healing, and 2 (14.3%) experienced therapeutic failure; one was treated with meglumine antimoniate, and one was treated with miltefosine. In the control group, 66 patients were treated with meglumine antimoniate, two were treated with ketoconazole, and in two patients, the treatment regimen was not stated in their clinical records. Among the controls, 44 showed skin lesion healing, while 21 (30.1%) patients experienced therapeutic failure; from those, 20 were treated with meglumine antimoniate and 1 with ketoconazole. There was no information regarding the treatment outcome in three patients from the control group treated with meglumine antimoniate. No statistical differences were found between cases and controls in CL therapeutic failure to meglumine antimoniate (p=0.211)."

Discussion

Line 262: "there is not sufficient knowledge to guide clinical decisions…" The authors need to specify on which aspects they are referring to here.

- ⇒ A sentence was introduced in lines 302-304 to expand that affirmation. "but there is not sufficient knowledge yet to guide clinical decisions or well-defined prevention strategies, such as early markers of progression from the CL to the ML form of the disease."

Line 274: "are highly related" – do you mean here "phylogenetically close"?

- ⇒ Yes, that is exactly what we mean. To clarify that, the paragraph was modified in line 319: "Nevertheless, it is essential to note that most patients were infected with parasites from the *Leishmania* (*Viannia*) subgenus, which are closely related species."

Regarding the documented demonstration of LRV1 presence in L. panamensis, there is a recent study performed in Panama (Gonzalez et al., 2023), which did not find support for a significant association of

LRV1 presence in cultured parasite isolates with disease severity in human leishmaniasis. This needs to be discussed in the manuscript.

⇒ The reference was included as number 39 and discussed in lines 332-335 as follows:
"A recent study analyzed 56 *L.* (*V.*) *panamensis* isolates from cutaneous lesions in Panama from 2014-2018 and found that 20% were infected with LRV1[39]. This contrasts with the observation in Colombia, where around 170 isolates of *L.* (*V.*) *panamensis* collected between 1983 and 2021 were found to have none infected with LRV1[40]. It's worth noting that none of the referenced studies involved *L.* (*V.*) *panamensis* isolates from ML patients. However, in the analysis presented here, *L.* (*V.*) *panamensis* was found in samples from both groups (cases, 18.1%, and controls, 15.7%), and the only two biopsies infected with *L.* (*V.*) *panamensis* carrying LRV1, belonged to the ML group. Taken together, these observations, along with the fact that most of the samples in this study came from the Amazon, Orinoco, and central and east Andean Colombian regions (Figure 1), suggest that the differential geographic distribution and frequencies of LRV1 could be indicative of different parasite gene flows associated with each environment [41]."

Line 297: "also due to study design disparities" – I suggest to add here "including varying lengths of patient follow-up"

⇒ Modification is done in line 358.

The discussion section would benefit from a deeper discussion of the findings of this study and the perspectives for future work. For instance:

(related to lines 324-328): It would be interesting to prospectively follow up CL cases from whom LRV-positive parasites were isolated, and establish whether or not the presence of the virus increases the risk of complications. It would be relevant that such a study assesses the LRV load variability among the LRV+ tested biopsies and if there is a higher Leishmania parasite load in lesions which are due to LRV+ parasites.

Based on published work and the findings of present work, the authors need to elaborate on how a complete treatment for a previous CL episode has a protective role in the occurrence of ML.

The wide 95% confidence interval associated with the OR estimate for LRV1-ML (in the multiple logistic regression analysis) in this study means the sample size is small (as acknowledged by the authors in the discussion section). This does not mean the biological effect is not real. The authors should emphasize this further in the discussion by stating that a bigger sample size is required (instead of "may be required", line 303) to have a more accurate effect size estimate of the association between LRV1 presence and disease severity (ML presentation) in human tegumentary leishmaniasis.

⇒ The discussion was written again following the referee's suggestions.
The main changes in the discussion from line 354 are on,

- The implications of the completed and systemic treatment for the CL event in preventing ML occurrence.
- The perspectives for future studies for assessing LRV1 detection and load calculations as prognostic markers in CL through prospective studies.
- The current study's limitations.

About the observation in line 303, this was amended in lines 362-364 as follows:
"However, the wide confidence interval of the odds ratio (OR) linking LRV1 with ML observed in the present study indicates that a larger sample size is needed to achieve greater precision in this association."

Other points:

Scientific names are written in italics. Please revise this throughout the text, including Tables and Figures.
⇒ Modifications were made in the new discussion and the supplementary tables.

The standard nomenclature for the Leishmania subgenera is as follows: Leishmania (Viannia) subgenus
⇒ Modification is done on lines 30 and 319.

Introduction, lines 60-61, "Pentavalent Antimonial" > pentavalent antimonial
⇒ Modification is done in line 62.

Introduction, line 63 & Methods, line 139, "gender" > sex
⇒ Modification is done in line 64.

Introduction, line 63, "immune polymorphism" > immune response gene polymorphism
⇒ Modification is done in line 64.

Methods, line 174: specify that OR means odds ratio
⇒ Modification is done in line 182.

Results, line 250: "protector" > protective
⇒ Modification is done in line 290

Discussion, line 287: "duplet" > duet or couple
⇒ The sentence was removed from the manuscript.

Figure 1 labels: please replace L. pana/guyanensis with L. panamensis /guyanensis

⇒ Modification is done in line 278 and in the figure.

Supplementary Figure S1: 18S gene > 18S rDNA gene

Supplementary Figure S1: box corresponding to the control group definition. “Meets criteria for case definition (Total n=70)” should be replaced with “Meets criteria for control definition (Total n=70)”

⇒ Corrections were made.

Supplementary Table 2:

-Replace “undeterminated” with “undetermined” (meaning absent Ct values)

-Verify the correct name RPL27 (in some instances it was written as RLP27)

Supplementary Table 2 caption:

-replace “in order to associated them” with “to associate them”

-replace “subtalbe” with “subtable”

⇒ All corrections were made in Supplementary Table 2.

Reference 40 appears together with ref. 39, thus it gets unnoticed.

⇒ The references were amended.

<u>Reviewer #2.</u>

Line 58 and 273: Correct "Leishmania Viannia" to "Leishmania (Viannia)"

⇒ Correction in lines 30, 59, 417

Line 323: Correct "Leishmania (V.)" to "Leishmania (Viannia)" or "L. (Viannia)"

⇒ Correction in lines 30, 59, 417

Methodology / Results

Some steps of the methodology need better explanation:

Frozen biopsies maintained at −80 °C were processed to confirm infection by Leishmania spp. and LRV1 detection: Were the biopsy samples frozen at -80°C without any preservative?

⇒ Yes. to state that “Dry” was introduced in line 153.

For the cases, were mucosal lesion biopsy samples used, and for the controls, cutaneous lesion samples?

⇒ Yes. It was clarified in lines 153-154.

1. Leishmania spp. Infection was confirmed by either RT qPCR or qPCR of the 18S ribosomal subunit gene of the parasite: Were all 103 samples subjected to both methodologies? If so, what were the results?

⇒ In the supplementary figure 1 of the evaluated manuscript, it is presented that the *Leishmania* spp. 18S ribosomal gene was detected in the cDNA in 45 controls and 19 cases, as well as in the DNA of 25 controls and 14 cases. However, a new supplementary figure (Supplementary Figure 1.B) was introduced, presenting the step-by-step Lab. procedure to clarify it. Cts obtained for the 18S ribosomal subunit gene are presented in the supplementary Table 3.

Why was the parasite load not quantified, as this could allow for an assessment of whether the presence of LRV1 is associated with parasite load and whether this is associated with the mucosal form of leishmaniasis?

⇒ Parasite load was not quantified because this work aimed to determine if the presence of LRV1 was somehow associated with the occurrence of LRV1. Parasite detection was part of the algorithm to assess what samples were suitable for LRV1 detection. Given that we were using fragments of cryopreserved biopsies without adjusting by tissue weight, we could not compare the parasitic load between samples. The referee is right that it will be desirable to adjust the LRV1 detection according to the parasite load in the clinical sample. That procedure must be standardized for future studies where the researchers control tissue sampling.

The reference cited in this section is from the group, but it references the work of van den Bogaart et al. 2013 (doi: 10.1016/j.ijpddr.2013.11.001); this study uses the human β2-microglobulin (β-2M) mRNA gene, so it would be important not only to cite the original references but also to mention whether there were any modifications.

⇒ The referee is right about the primers for *Leishmania* spp. 18S ribosomal gene are derived by the protocol described by den Bogaart et al. 2013. However, we performed a completely different protocol described in our published paper, so we cite that work. In the work cited, we properly refer to the primers as the ones designed by den Bogaart et al. 2013. In the procedure performed and described in the cited reference, we did not detect the β2-microglobulin (β-2M) nor run the duplex q-RT-PCR.

It would be important to know if an endogenous control was used and, if so, what the results were.

⇒ It is not clear what the referee means by endogenous control. If this is regarding the quality of the RNA, the endogenous control used to assess the cDNA quality was the human gene, rpl27, to avoid false negatives associated with long-term storage samples. As stated in the lines 161-163 and the results presented in Supp Table 2.
"cDNA quality was assessed via RT-qPCR of a human gene, encoding the ribosomal protein L27 (RPL27) [32], in order to avoid false negatives associated with long-term storage samples, following the algorithm presented in supplementary Fig.1. B. "

⇒ Additionally, the step-by-step lab procedure description was introduced as supplementary Figure 1. B.

It's also crucial to know if the samples that could not have the Leishmania species identified were positive in these parasite detection assays and just couldn't be identified or if they were negative and the endogenous control was positive.

⇒ Samples negative for *Leishmania* spp. 18S ribosomal gene in cDNA and DNA and samples negative for the human gene rpl27 in cDNA were excluded from the study, as presented in supplementary figure 1. B.

2. Infecting parasite species were identified via PCR amplification of the miniexon gene [33] and PCR-RFLP of the hsp70 gene using as controls MHOM/BR/75/M4147, MHOM/PA/71/LS94, MHOM/BR/75/M2903, MHOM/BZ/82/BEL 21 and MHOM/BR/73/M2269 [34]: Here again, it would be better to cite the original references for PCR for the miniexon and hsp70 genes. In this sense, I ask, and I think it's important to add the answers to the article, if all samples were subjected to both protocols, or if one protocol was done and only the negative samples or those that couldn't have the Leishmania species identified were subjected to the other protocol.

It's also noteworthy that the cited PCR hsp70 protocol (Garcia et al. 2004) is very good for Leishmania in culture, but it is known not to have good sensitivity for detecting Leishmania directly in clinical samples, likely due to the long PCR fragment amplified. However, there are other approaches that improve the detection and identification capacity of Leishmania spp., which could be used to improve the quality of the results, These approaches are already very good but have a little over 20% of samples without species identification. This identification seems quite relevant since the presence of LRV1 appears to be more frequent in L. guyanensis and L. braziliensis (from Amazonian areas) than in L. panamensis, a species quite common in Colombia. Figure 1 seems to show that among the samples without species identification, a higher number of LRV1+ samples are in the "cases" group than in the "control" group, and the negatives are more frequent in the "control" group. If we consider that LRV1 is not so frequent in L. panamensis and that the mucosal form should be more associated with L. braziliensis, it would be important to identify as many samples as possible.

⇒ Supplementary Table 3 was introduced to clarify how many samples were submitted to each of the procedures for identifying the Leishmania species described in the paper.

Regarding the references, we identify the species using a nested hsp-70 PCR. A protocol developed for us aimed to increase the test's sensitivity when using biopsies or stained smears. Therefore, the protocol's reference is the one that is cited in the manuscript corresponding to a paper published for us in 2016 (doi: 10.1128/JCM.02955-14). In that reference, we gave the proper credit to the original and wonderful protocols designed by Garcia et al. 2004 (doi: 10.1128/JCM.42.5.2294-2297.2004) and Montalvo et al. 2010 (doi: 10.1017/S0031182010000089).

It is unclear what the referee means by referencing Garcia et al. (2004). We did not cite that work in the paper precisely because this protocol has low sensitivity with clinical specimens.

The referee is absolutely right that it would be desirable to increase the samples with Leishmania species identification; we made several attempts to achieve that. However, given that parasite

species is not the focus of the work, nor is it to identify the frequency of the LRV1 regarding the species, we had to stop there to prioritize Lab. resources and sample availability for the LRV1 detection.

3. Supp. Fig2: The regions cited in Supp. Table 1 (Orinoco, Amazon, Pacific, Andean) could be presented: it is interesting to see that the occurrence of LRV1-containing parasites seems to be concentrated in the central region of the country, between the Andean, Orinoco, and Amazon regions. I understand that this is not the focus of the study, but it seems like something interesting to comment on, especially since this is presented later in the discussion.

⇒ Figure 1 was modified to make that information more visible, and the paragraph in lines 256-266 was expanded to emphasize this data. However, as stated in lines 342-343, this study analyzed samples from the Amazon, Orinoco, and Andean regions. A study to understand the geographical distribution and frequency of LRV1 in our country will need a proper design.

4. Line 300: Include the mentioned reference regarding the study by Cantanhede et al.

⇒ Correction made in line 348.

<u>Reviewer #3</u>:

Animal studies have shown definitively that the endobiont dsRNA virus LRV1 is associated with increased pathology and metastasis in animal models. While not strictly equivalent, this has prompted many to extrapolate that in humans, the presence of LRV1 may be associated with increased risk of mucocutaneous leishmaniasis. However, various clinical surveys or retrospective studies have yielded variable results, with some showing increased association of ML with LRV1 and some not. The reasons for this are unknown and given the paucity of data and importance of this question for treatment options, additional studies are needed. This work addresses this nicely, with carefully presented analysis and data.

The authors report that in their study site (Colombia) there is a significant elevation of the risk of ML with the presence of LRV1. I was not entirely sure about some of the authors statements which to me imply that the difference is 26% rather than 16% (example from abstract: Results: The overall frequency of LRV1 was 16.5 (95% CI,10.4 – 25.12) higher in samples from cases than controls [33.33 % (95% CI,18.89 – 51.76) vs 8.57% % (95% CI, 3.82 – 18.10]. So this should be clarified but as shown in a table, this is statistically significant. This could be more clearly stated and emphasized in the abstract and discussion.

⇒ The abstract was corrected in lines 37-39, affirming that:
"The frequency of LRV1 in the 103 patients was 16.5% (95% CI,10.4 – 25.12) being higher in samples from cases [33.33 % (95% CI,18.89 – 51.76) than from controls [8.57% (95% CI, 3.82 – 18.10)]."

⇒ In lines 220-222, a paragraph was introduced stating: “In the bivariate analysis presented in Table 1, the variables significantly differing between cases and controls are the completed treatment for the CL event and carrying a parasite infected with LRV1.”
In lines 288-289, it is stated that “The multivariate analysis presented here on 103 patients (CL,70 and ML, 33) indicates that contracting an infection with Leishmania spp.-LRV1(+) is independently associated with ML”.

Another important finding is that failure to complete a treatment course against CL resulted in increased ML likelihood. Data supporting This seemingly obvious finding is in fact difficult to find in the literature and deserves more emphasis - as to me it is understated. Besides the health implications, this may be one factor clouding the assessment of ML-LRV1 associations, as in effect some patients with LRV1 and destined for ML are instead counted as 'not associated'. This has been suggested in several papers that could be cited but this is the first evidence to my knowledge.

This finding as well the reports that exogenous viral infections also can stimulate metastasis in animal models could explain some of the diverse reports for against ML-LRV1 association, and perhaps this could be briefly discussed.

⇒ The discussion was written again, trying to expand the central point cited by the referee and incorporating new references. Please see the amendments.
in lines 366-399,
“Moreover, the multivariate analysis revealed that completing the treatment for CL significantly reduced the risk of developing ML [OR, 0.039 (95% CI, 0.01-0.12); $p < 0.0001$]. Although this study did not follow a prospective design to directly assess the impact of completed CL treatment on preventing ML, the results are consistent with previous findings [42-43]. They support the long-held but unproven assumption that systemic treatment of CL with the appropriate dosage and duration can effectively prevent ML [44,45]. The systemic versus local treatment of New World CL is a hot topic of discussion. One of the main arguments supporting local therapies is based on reports of viable *Leishmania* parasites in the original cutaneous scars and the intact nasal mucosa of patients after completing systemic treatment with highly toxic medications [46,47]. Additionally, even in the present study, it has been observed that some patients adequately treated for CL may still develop ML.

Experimental evidence has shown that Leishmania spp. carrying LRV1 can lead to complications such as relapse and parasite metastasis [19,20]. The authors of observational studies showing LRV1's association with therapeutic failure in CL treatment with antimony and pentamidine have suggested that LRV1-mediated changes in the human host immune response potentially affect the efficacy of drugs [22,23]. It is well established that the host immune system plays a crucial role in the response to treatments [48-50]. Therefore, the mechanisms involving each of the variables associated with ML occurrence are likely rooted in the background of the host immune system, and their exact contributions to the multifactorial occurrence of ML may be challenging to assess.”

And in lines 422-425:
"A randomized controlled trial to assess the effectiveness of CL treatment in preventing ML occurrence is not ethically suitable. Therefore, the role of this factor will continue to be a subject of debate until a better understanding of the immunological factors mediating the CL response to treatment is achieved."

Maria Clara Echeverry G.
