# Supplementary material for: Mucosal leishmaniasis is associated with the Leishmania RNA virus and inappropriate cutaneous leishmaniasis treatment
Source: PLoS One. 2025 Jan 24;20(1):e0317221. doi: 10.1371/journal.pone.0317221 (PMC11759362; doi:10.1371/journal.pone.0317221)
Supplement: Attachment — Submitted filename: Response to Reviewers.docx [file pone.0317221.s008.docx]

Dear Editor and Reviewers,

Please find below the response to each one of your observations.

Journal Requirements:

Please review your reference list to ensure that it is complete and correct. If you have cited papers that have been retracted, please include the rationale for doing so in the manuscript text, or remove these references and replace them with relevant current references. Any changes to the reference list should be mentioned in the rebuttal letter. If you need to cite a retracted article, indicate the article’s retracted status in the References list and also include a citation and full reference for the retraction notice.

- All the references have been reviewed and adjusted according to Plos One instructions.

Changes were introduced in references 6, 13, 14, 29,30,31, 42 and 43.

Reference number 29 was corrected, given that this reference was amended in 2016. The erratum was incorporated into the citation according to what appears in PubMed Central. The amendment concerned the probe sequence they presented in 2014 for detecting the human B-2 microglobulin gene. However, our work did not involve the detection of that gene. Therefore, to make clear that we use just the primers for the *Leishmania* spp 18S ribosomal RNA gene published in that paper, but that the method is the one described previously by us in reference number 30, the sentence on lines 120-121 was amended to “to establish the presence of *Leishmania* spp., via qPCR for the 18S ribosomal RNA gene the primers referred by van den Bogaart E [29] were used following the protocol modified as described [30].”

Responses to Reviewers

Reviewer #1: In this revised version of the manuscript, the authors have significantly improved the manuscript and adequately addressed my concerns. The paper is ready for publication with a few editions in the text to be incorporated, as pointed out below.

Methods, line 114: regarding the “therapeutic test”. For me the clarification provided by the authors does not correspond to a test of cure; rather, it corresponds to the definition of the therapeutic clinical outcome (i.e. clinical cure) at the time point of follow-up assessment (one month after treatment). This needs to be revised in the manuscript.

- The correction was made on lines 114-115, stating that it corresponds with “clinical response to treatment.”

Discussion, line 381: please replace “parasitic” with “parasite” (i.e. parasite load estimation)

- The correction was made on line 381

Scientific names should be written in italics; some instances need to be amended, please revise this thoroughly.

- Changes were made in Table 1. Lines 498, 502 and 503.

Kind Regards,

Maria C. Echeverry G.

Response to the requirement made on Dec 1, 2024

Thank you for your email and for pointing out the issues regarding Figure 1.

We have updated the map, changing the natural regions layer. We introduced that change because we couldn’t assess the copyright restrictions of the source we used in the previous version of the map. Therefore, we generated our layer. The figure caption was updated in the manuscript body; all the files were updated in the “Editorial Manager.”

Answering your questions:

a) Where did the authors obtain the map in Figure 1

- The map in **Figure 1** was created using **QGIS 3.38.3** (<https://qgis.org/license/>).

**Data Sources**:
The layers used to produce the map were obtained from publicly available and open-access sources, as outlined below:

- **Ocean layer**: Natural Earth Data - 10m Physical Vectors
  <https://www.naturalearthdata.com/downloads/10m-physical-vectors/10m-ocean/>
  Terms of Use: <https://www.naturalearthdata.com/about/terms-of-use/>
- **Elevation layer**: DIVA-GIS - Elevation Data
  <https://diva-gis.org/data.html>
  About DIVA-GIS: <https://diva-gis.org/index.html>
- **Colombia administrative boundaries**: GADM - Download Country Data
  <https://gadm.org/download_country.html>
  About GADM: <https://gadm.org/about.html>

**Note:** The natural regions layer shown on the map is our authorship and was generated by modifying the departmental boundaries layer obtained from GADM.

b) Please state whether the map has been previously copyrighted to your knowledge.

To the best of my knowledge, this map has not been previously copyrighted. The data sources referred are free to use for publication under open-access conditions and do not impose copyright restrictions that conflict with the **CC BY 4.0 license**.

If you need further clarification or additional information, please do not hesitate to let me know. I will respond promptly.

Thank you for your time and consideration.

Maria C. Echeverry
